# Summer Reading

**Published:** 1858-07

**Authors:** 


SUMMER READING.—No physician can do better, with as little money, than to purchase *Braithwaite's Retrospect* for June, of STRINGER & TOWNSEND, for one dollar, or *Ranking's Abstract*, at the same price, from LINDSAY & BLAKISTON, of Philadelphia, both containing the cream of medical progress at home and abroad for the previous six months; then the sterling quarterly, the *Medico-Chirurgical Review*, republished by the Messrs. WOOD, of New York, at $3 a year. For raciness, and practical good sense on subjects pertaining to health, *Dixon's Scalpel* has no equal—a quarterly number for twenty-five cents. We looked over an article in *Graham's Magazine* for July, with unusual interest, having been written by a Doctor of Divinity in fifteen hundred and nine. By a singular coincidence, GODEY too, has been delving into the musty records of the past, and gives us nineteen plates of cooking utensils used in seventy-nine; his July frontispiece, "Worship in the Wilderness," may be read by the hour with absorbing interest—for pictures may be read. *Arthur's Home Magazine* lags, but it will be good when it comes. *The Gymnast*, by S. J. SEDGWICK, 95 Sixth Avenue, where the youth of both sexes may develop mind as well as muscle, by the practice of those agilities which are admirably adapted to the promotion of physical vigor. Every family in a city ought to patronize a well-ordered gymnasium.

